# Biosynthesis of cofactor‐activatable iron‐only nitrogenase in *Saccharomyces cerevisiae*

**DOI:** 10.1111/1751-7915.13758

**Published:** 2021-01-28

**Authors:** Gema López‐Torrejón, Stefan Burén, Marcel Veldhuizen, Luis M. Rubio

**Affiliations:** ^1^ Centro de Biotecnología y Genómica de Plantas Universidad Politécnica de Madrid (UPM) Instituto Nacional de Investigación y Tecnología Agraria y Alimentaria (INIA) Pozuelo de Alarcón, Madrid 28223 Spain; ^2^ Departamento de Biotecnología‐Biología Vegetal Escuela Técnica Superior de Ingeniería Agronómica Alimentaría y de Biosistemas UPM Madrid Spain

## Abstract

Engineering nitrogenase in eukaryotes is hampered by its genetic complexity and by the oxygen sensitivity of its protein components. Of the three types of nitrogenases, the Fe‐only nitrogenase is considered the simplest one because its function depends on fewer gene products than the homologous and more complex Mo and V nitrogenases. Here, we show the expression of stable Fe‐only nitrogenase component proteins in the low‐oxygen mitochondria matrix of *S. cerevisiae*. As‐isolated Fe protein (AnfH) was active in electron donation to NifDK to reduce acetylene into ethylene. Ancillary proteins NifU, NifS and NifM were not required for Fe protein function. The FeFe protein existed as apo‐AnfDK complex with the AnfG subunit either loosely bound or completely unable to interact with it. Apo‐AnfDK could be activated for acetylene reduction by the simple addition of FeMo‐co *in vitro*, indicating preexistence of the P‐clusters even in the absence of coexpressed NifU and NifS. This work reinforces the use of Fe‐only nitrogenase as simple model to engineer nitrogen fixation in yeast and plant mitochondria.

## Graphical abstract

Fe‐only nitrogenase components were expressed and matured in *S. cerevisiae* mitochondrial. The electron donor component was active as isolated and the catalytic component was ready for activation by cofactor insertion.

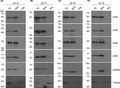

## Introduction

Biological nitrogen fixation (BNF), the reduction of dinitrogen (N_2_) to ammonia (NH_3_), is catalysed by the nitrogenase protein, an O_2_‐sensitive metalloenzyme complex present in some prokaryotes called diazotrophs (Raymond *et al*., 2004). Three variants of nitrogenase exist in nature and are defined as the molybdenum (Mo), vanadium (V) or iron‐only (Fe‐only) nitrogenase with respect to their cofactor metal composition. All diazotrophs carry a Mo nitrogenase and some of them additionally carry alternative V or Fe‐only nitrogenase (Seefeldt *et al*., 2009; O’Carroll and Santos, 2011). The Mo nitrogenase is the best studied one and consists of an Fe protein (NifH) homodimer acting as electron donor and a MoFe protein (NifDK) heterotetramer that is the catalytic component. The electrons provided by NifH accumulate at the iron–molybdenum cofactor (FeMo‐co) of NifDK where N_2_ is reduced (Seefeldt *et al*., 2009). The V and Fe‐only nitrogenases also have two components, the Fe protein (AnfH) and FeFe protein (AnfDGK) for the Fe‐only nitrogenase, and the Fe protein (VnfH) and FeV protein (VnfDGK) for the V nitrogenase, but much less is known about their biosynthesis and catalytic properties. Of the three, the Fe‐only nitrogenase is considered as the simplest one because its function depends on fewer gene products than the homologous and more complex Mo and V nitrogenases (Joerger *et al*., 1989).

Despite BNF contributions, most reactive N in agricultural managed ecosystems is of industrial origin (Tilman *et al*., 2011; Foley *et al*., 2011). To reduce dependency of synthetic N fertilizers, mainly in cereal crops, researchers are trying to leverage biotechnology. One proposed strategy involves direct transfer of prokaryotic nitrogenase genes into cereal plants (Vicente and Dean, 2017; Burén and Rubio, 2018). In this path, *Saccharomyces cerevisiae* stands as unicellular, easy to culture, genetically tractable, model of eukaryotic cell. Expression of active NifH (the Fe protein component of the Mo nitrogenase) and NifB (a FeMo‐co maturating protein) in mitochondria of *S*.* cerevisiae* has proven that these extremely O_2_‐sensitive nitrogenase proteins can accumulate in a eukaryotic cell, representing the first steps towards engineering nitrogenase in eukaryotes (López‐Torrejón *et al*., 2016; Burén *et al*., 2017). Recent studies have also shown that both mitochondria and chloroplasts of tobacco can harbour active NifH at the end of the dark period (Eseverri *et al*., 2020; Jiang *et al*., 2021).

Engineering the Fe‐only nitrogenase is attractive because of its genetic simplicity and because availability of Mo (and V) in mitochondria and chloroplasts is not well understood. In the diazotrophic bacterium *Azotobacter vinelandii,* the Fe‐only nitrogenase genes are organized in a single *anfHDGKOR* gene cluster (*anf* for alternative nitrogen fixation genes) (Joerger *et al*., 1989). The *anfH*, *anfD* and *anfK* genes are equivalent to their Nif counterparts, whereas the *anfG* gene encodes a gamma subunit, as in contrast to the Mo nitrogenase NifDK protein the alternative nitrogenases exist as heterohexamers (VnfDGK and AnfDGK) in their mature forms. The structure of the homologous VnfDGK component of the V nitrogenase has been solved (Sippel and Einsle, 2017). While the active‐site metallocluster FeFe‐co is thought to be structurally and functionally equivalent to FeMo‐co, the N_2_ reduction activity of Fe‐only nitrogenase is about three times lower (Krahn *et al*., 2002; Harris *et al*., 2018). Genetic evidence indicates that the product of the NifB enzyme (a [Fe_8_S_9_C] cluster named NifB‐co) serves as biosynthetic precursor for all three nitrogenase cofactors FeMo‐co, FeV‐co and FeFe‐co (Bishop and Joerger, 1990; Curatti *et al*., 2007). Genetic and transcriptome analysis of *A*.* vinelandii* has suggested that the Anf system depends on VnfEN for FeFe‐co maturation (Wolfinger and Bishop, 1991; Hamilton *et al*., 2011). However, work by Yang and colleagues later showed that neither NifEN nor VnfEN scaffold proteins were required for the biosynthesis of FeFe‐co in *Escherichia coli* (Yang *et al*., 2014 *)*, although they are essential for the biosynthesis of FeMo‐co (Jacobson *et al*., 1989) and FeV‐co (Wolfinger and Bishop, 1991) respectively. This is consistent with studies showing that several diazotrophs lack the NifEN protein (Soboh *et al*., 2010) and that FeFe‐co biosynthesis can take place in its absence (Schüddekopf *et al*., 1993).

In addition to the structural components, nitrogenase expression and maturation in native hosts and heterologous systems require several gene products with accessory functions. In *E*.* coli*, an engineered 10‐gene cluster (*anfHDGK*, *nifBUSV*, *nifF* and *nifJ*) generated active Fe‐only nitrogenase (Yang *et al*., 2014 *)*. The products of *nifJ* and *nifF* are involved in electron donation to nitrogenase, whereas the *A*.* vinelandii anfO* and *anfR* genes did not seem to be strictly required. In yeast, the [Fe–S] cluster assembly proteins NifU and NifS were not necessary to produce active NifH in mitochondria (López‐Torrejón *et al*., 2016), suggesting that the mitochondrial [Fe–S] cluster biosynthetic machinery of yeast could support [Fe_4_S_4_] cluster insertion into NifH. On the contrary, NifU and NifS were essential to obtain NifB with a complete set of [Fe_4_S_4_] clusters in the very same system (Burén *et al*., 2019), emphasizing that exact genetic requirements differ among nitrogenase proteins.

In this work, the structural components of the *A*.* vinelandii* Fe‐only nitrogenase (AnfH and AnfDGK) were expressed together with the [Fe–S] cluster biosynthetic proteins NifS and NifU in *S*.* cerevisiae* and targeted to the mitochondrial matrix. Purified yeast AnfH was active as Fe protein, and enriched preparations of yeast AnfDK could be activated *in vitro* by the simple addition of purified FeMo‐co. Interestingly, cofactor‐deficient apo‐FeFe protein was a heterotetramer lacking the AnfG subunit. This is the first report of an engineered eukaryotic nitrogenase catalytic component ready for activation by cofactor insertion.

## Results

### Expression of Fe‐only nitrogenase structural components in *S*.* cerevisiae*

**Fig. 1:**
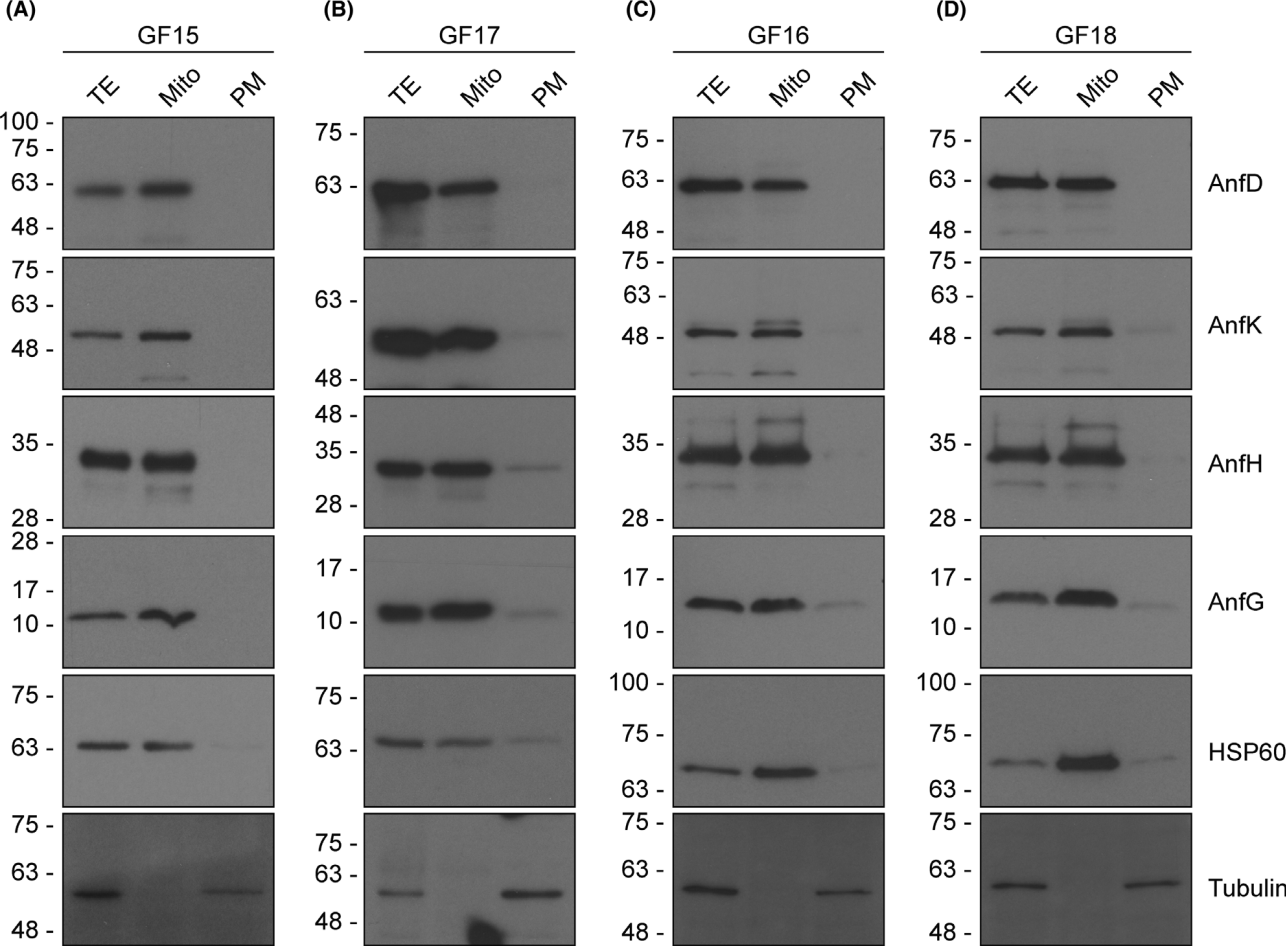
Expression and mitochondria targeting of nitrogenase proteins in *S*.* cerevisiae*. Immunoblot analysis of yAnfD, yAnfK, yAnfH and yAnfG proteins in total protein extracts (TE), in isolated mitochondria (Mito) and in ‘post‐mitochondrial supernatant’ fractions (PM) from *S*.* cerevisiae* strains GF15 (A), GF17 (B), GF16 (C) and GF18 (D). Total extracts and mitochondria isolation were prepared from aerated cultures of galactose‐induced cells. Antibodies recognizing *A. vinelandii* AnfD, AnfK, AnfH and AnfG proteins, HSP60 (mitochondrial marker protein), or tubulin (cytosolic marker) were used for immunoblotting analysis.

To synthesize Fe‐only nitrogenase components in *S*.* cerevisiae,* we generated strains expressing the *A*.* vinelandii anfHDGK* genes either alone or in combination with the [Fe–S] cluster assembly proteins NifU and NifS (Table 1 and Table S1). All genes were synthesized with codon optimization for *S*.* cerevisiae* (Fig. S1), cloned into pESC expression vectors containing the galactose‐regulated GAL1 and GAL10 promoters, and introduced into *S*.* cerevisiae* W303‐1a by transformation. Nitrogenase gene expression was induced under respiratory growth conditions with galactose as carbon source. To ensure mitochondrial protein targeting, the *su9* leader sequence (Westermann and Neupert, 2000) was fused to the 5′‐end of each *anf* and *nif* gene sequence (Fig. S2). Correct translocation of each expressed component to the organelle was verified by SDS‐PAGE and immunoblot analysis of isolated mitochondria (Fig. 1). All Anf proteins were specifically targeted to the mitochondria, while a fraction of NifU and NifS proteins could also be found in the extra‐mitochondrial fraction. Fe‐only nitrogenase proteins were detected using antibodies generated to specifically recognize the AnfH, AnfD, AnfK and AnfG polypeptides. For this, AnfH, AnfD, AnfK and AnfG were individually expressed and purified from recombinant *E*.* coli* cells (Fig. S3).

**Table 1:** *S. cerevisiae* strains expressing combinations of Anf and Nif proteins.

Strain	Proteins expressed	Purification tags present
GF13	AnfH, NifUS	His_10_‐AnfH
GF15	AnfHDGK	His_10_‐AnfH, His_10_‐AnfD
GF16	AnfHDGK, NifUS	His_10_‐AnfH, His_10_‐AnfD
GF17	AnfHDGK	His_10_‐AnfH, TS‐AnfD
GF18	AnfHDGK, NifUS	His_10_‐AnfH, TS‐AnfD
GF19	AnfHDGK	His_10_‐AnfH, SS‐AnfD
GF20	AnfHDGK, NifUS	His_10_‐AnfH, SS‐AnfD

His_10_, 10 histidine tag at N‐terminal end; TS, Twin‐Strep‐tag at N‐terminal end, SS, Single Strep‐tag at the N‐terminal end.

### Isolation of yAnfH from mitochondria of *S. cerevisiae*

**Fig. 2:**
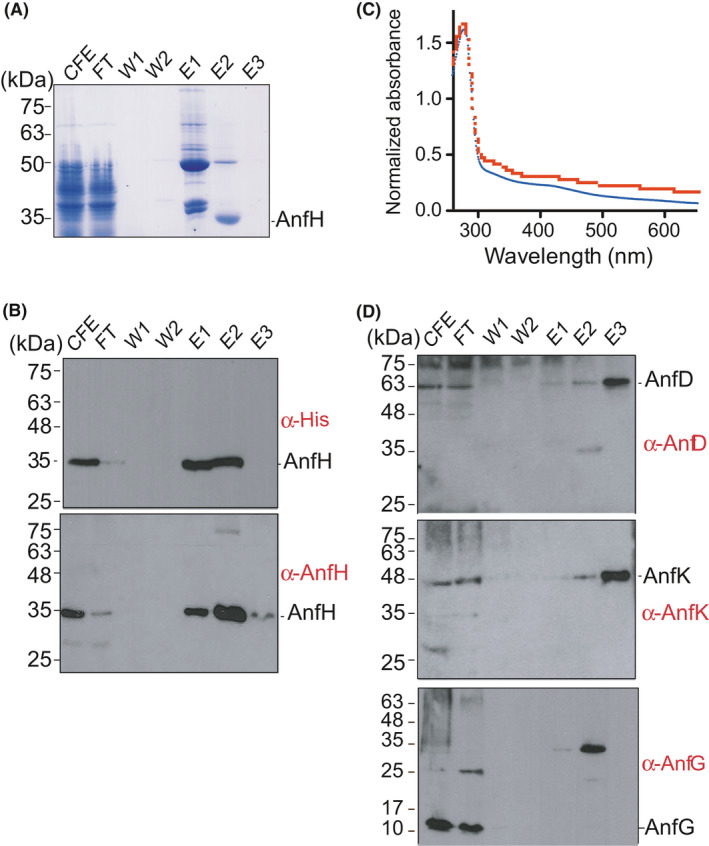
Purification of yAnfH from *S. cerevisiae* GF15. Coomassie‐stained SDS gel of purification fractions (A) and immunoblot analysis of the same fractions developed with antibodies against AnfH or the His‐tag (B), or with antibodies against AnfD, AnfG and AnfK (D). The 35 kDa band detected in E1 and E2 with α‐AnfG likely corresponds to unspecific recognition of an enriched polypeptide in yAnfH preparations. CFE, cell‐free extract; FT, flow‐through fraction; W_1_, fraction after washing with binding buffer; W_2_, fraction after washing with binding buffer supplemented with 80 mM imidazole; E_1_, E_2_ and E_3_, fractions eluted by applying a 100–175 mM imidazole gradient in binding buffer. C, UV‐visible absorption spectra of as‐isolated yAnfH (blue) and air‐exposed yAnfH (red).

Mitochondria‐targeted His‐tagged Fe protein (yAnfH) was purified by anaerobic Co^2+^ affinity chromatography from cells of GF13, GF15 and GF16 strains (see Table 1). The cultures were grown aerobically and induced as previously reported for the yNifH protein (López‐Torrejón *et al*., 2016). The yAnfH protein was purified to near homogeneity from cell‐free extracts of GF15 and GF16 strains, but it was poorly produced in GF13. SDS‐PAGE analysis of a typical yAnfH purification process from GF15 is shown in Fig. 2A. Most soluble proteins eluted at imidazole concentration between 100 and 130 mM (E1), while yAnfH eluted at 130 mM imidazole (E2). Immunoblot analysis developed with antibodies against the His‐tag or AnfH indicated that the majority of soluble yAnfH produced could be recovered during purification and that it migrated as expected with no proteolytic fragments present (Fig 2B). The yAnfH protein in purified preparations was quantified by BCA and by SDS‐PAGE band densitometry using ImageJ (Fig. S4A).

yAnfH exhibited the characteristic brown colour of nitrogenase Fe proteins. UV‐vis absorbance spectrum of as‐isolated yAnfH showed the 320 nm and 420 nm features associated with [Fe_4_S_4_] clusters (Fig. 2C) (Smith *et al*., 2005). Consistently, metal analysis confirmed the presence of 4 Fe atoms per yAnfH dimer. UV‐vis absorption spectral changes were observed upon exposure to air.

Because yAnfH was coexpressed with yAnfDGK in GF15 cells, the separation of both nitrogenase components during affinity chromatography was assessed by SDS‐PAGE and immunoblot. The yAnfDGK protein components were not detected by Coomassie staining (Fig. 2A). However, immunoblot analysis showed enrichment of AnfD and AnfK polypeptides in the E3 fraction containing 160 mM imidazole (Fig. 2D). The yAnfG protein was only detected in the flow‐through fraction, and therefore, it was neither stably associated with yAnfH nor with yAnfDK. The amount of yAnfDK protein in the preparations was quantified by immunoblot band densitometry using ImageJ against standards produced with *E*.* coli* purified AnfD and AnfK polypeptides (Fig. S4B).

### yAnfH functions as nitrogenase Fe protein

**Fig. 3:**
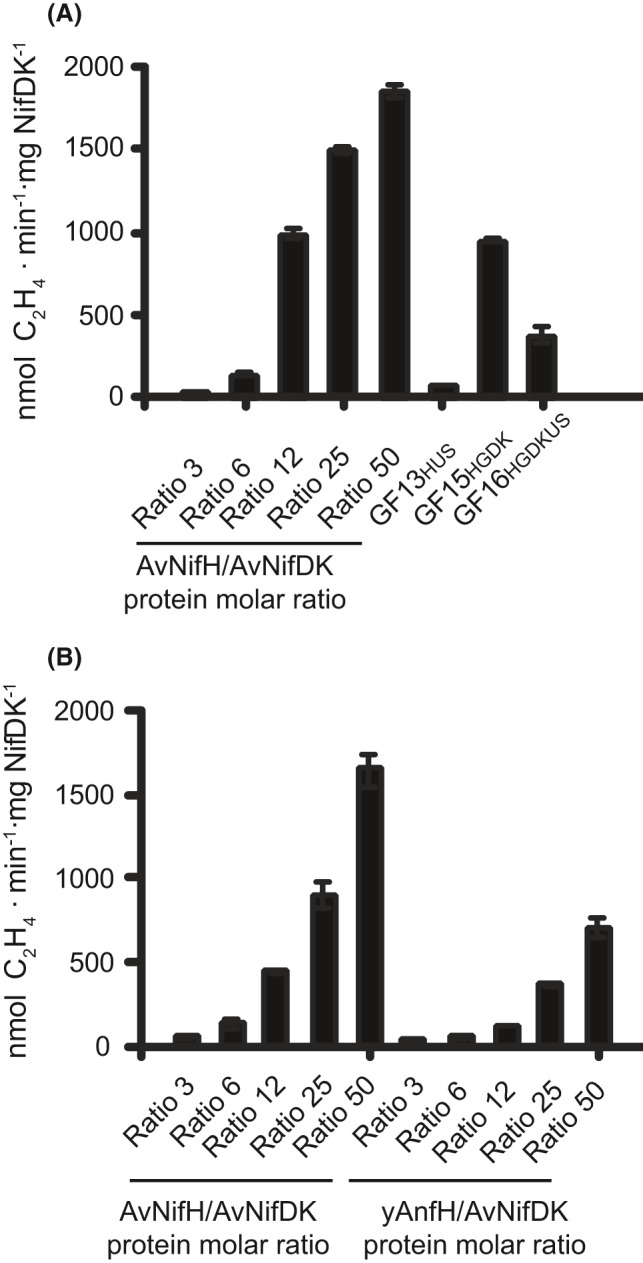
*Determination* of yAnfH *activity*. A. Determination of Fe protein activity in yAnfH purified preparations from strains GF13_HUS_ (coexpressing yAnfH with yNifU and yNifS), GF15_HGDK_ (coexpressing yAnfH with yAnfDGK) and GF16 _HGDKUS_ (coexpressing yAnfH with yAnfDGK, yNifU and yNiAS). B. AvNifDK activity titration with yAnfH purified from GF15. AvNifDK activity titrations with AvNifH were carried out as control reactions in (A) and (B). Data represent mean ± SD (*n* = 6) for each yAnfH containing reaction.

Fe protein function of as‐isolated yAnfH protein was tested using the acetylene reduction assay after addition of pure *A*. *vinelandii* MoFe protein (AvNifDK). The unavailability of *A*. *vinelandii* FeFe protein and the functional similarity between both nitrogenases (Schneider *et al*., 1997; O’Carroll and Santos, 2011; Harris *et al*., 2018) prompted us to use AvNifDK as complementary component. Electron donation to AvNifDK was observed, where the yAnfH protein isolated from the GF15 strain yielded NifDK specific activity of 950 units (nmol of ethylene formed·min^‐1^·mg of NifDK^‐1^) when yAnfH molar concentration was 100‐fold higher than AvNifDK (Fig. 3A). Surprisingly, activity was reduced by half when yAnfH was purified from GF16 cells coexpressing NifU and NifS. Furthermore, when yAnfH was coexpressed with NifU and NifS in the absence of yAnfDGK (strain GF13), activity was 10‐fold lower compared with GF16. One explanation for this observation could be that the presence of the yAnfDGK components could stabilize yAnfH, thereby improving its solubility or functionality.

Titration of AvNifDK activity with increasing amounts of yAnfH or AvNifH is shown in Fig. 3B. The specific activity of yAnfH was half compared to AvNifH along the titration consistent with a poorer interaction of AvNifDK with the non‐physiological complementary component. It is important to note that the assays in Fig. 3 compare yAnfH activity to that of AvNifH and not to the AvAnfH counterpart.

### Enriched preparations of yAnfDK from mitochondria of *S. cerevisiae*

**Fig. 4:**
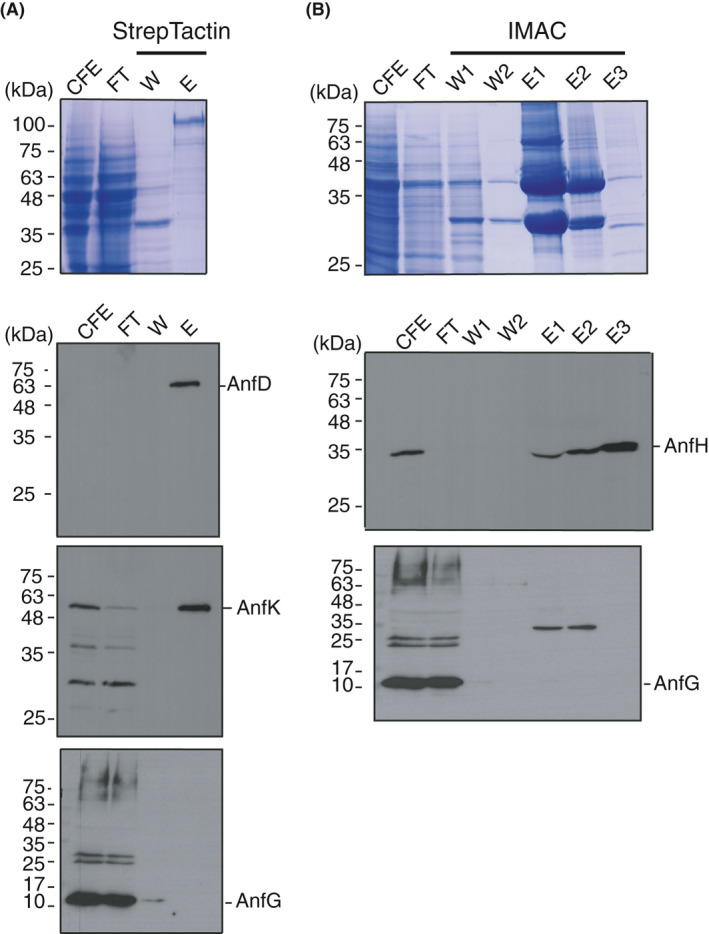
Enriched yAnfDK preparations from GF17 cells. A. Strep‐Tactin affinity chromatography fraction analysis by SDS‐PAGE (stained with Coomassie) and by immunoblots developed with antibodies against the AnfD, AnfK and AnfG polypeptides. CFE, cell‐free extract; FT, flow‐through fraction; W, fraction after washing with binding buffer; E, fraction after elution with 50 mM biotin in binding buffer. B. Analysis of purification fractions from Co^2+^ affinity chromatography (IMAC) by SDS‐PAGE (stained with Coomassie) and by immunoblots developed with antibodies against AnfH and AnfG. CFE and FT fractions as in panel (A); W_1_, fraction after washing with binding buffer; W_2_, fraction after washing with binding buffer supplemented with 80 mM imidazole; E_1_, E_2_ and E_3_, fractions after eluting with a 100‐175 mM imidazole gradient in binding buffer. The 35 kDa band detected in E1 and E2 with α‐AnfG likely corresponds to unspecific recognition of an enriched polypeptide in those fractions.

To improve yAnfDK protein isolation, the His‐tag at the N‐terminus of AnfD was replaced by either a Single‐ or Twin‐Strep‐tag. This strategy had previously been successful to purify NifB from yeast mitochondria (Burén *et al*., 2017b; Burén *et al*., 2019). Cells from aerated cultures of GF17 or GF19 strains coexpressing His‐tagged yAnfH were used as source of yAnfDK (Table 1). Protein preparations enriched in yAnfDK were obtained by anaerobic Strep‐Tactin affinity chromatography of soluble protein extracts, while yAnfH was simultaneously purified using anaerobic Co^2+^ affinity chromatography. A typical purification result from GF17 cells shown in Fig. 4. The enriched yAnfDK fraction showed light brown colour but the yAnfD and yAnfK polypeptides were not easily observed by Coomassie staining of SDS gels, although they were detected by immunoblot analysis using anti‐AnfD and anti‐AnfK antibodies (Fig. 4A). Again, yAnfDK co‐eluted as a complex devoid of yAnfG suggesting that yAnfG was either released during purification or that heterohexamer stability was low. The yAnfDK protein concentration in enriched fractions was estimated using ImageJ (Fig. S4B). Preparations of yAnfH protein purified by Co^2+^ affinity chromatography from GF17 soluble extracts contained more contaminants than those from GF15 cells (Fig. 4B).

### Activation of apo‐yAnfDK by isolated FeMo‐co

**Fig. 5:**
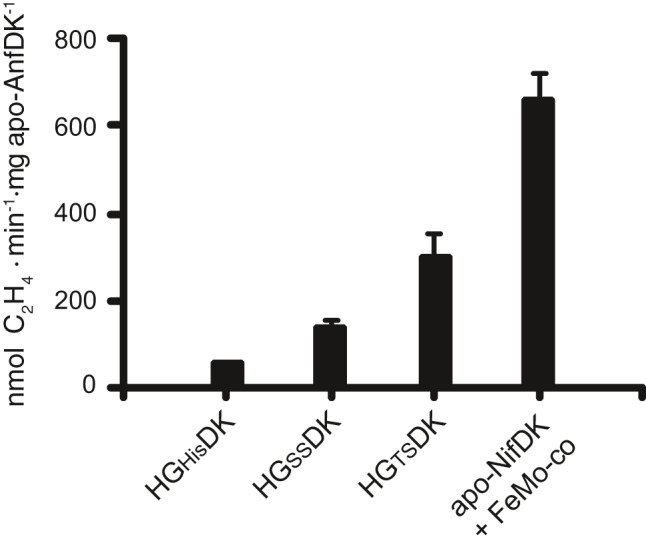
*In vitro* activation of apo‐yAnfDK using purified FeMo‐co. Preparations of apo‐yAnfDK with His_10_, Twin‐Strep (TS) or Single‐Strep (SS) purification tags at the N‐terminal end of AnfD were obtained from GF15 (expressing AnfHG_His_DK), GF17 (expressing AnfHG_TS_DK) or GF19 (expressing AnfHG_SS_DK) respectively. Five µl FeMo‐co isolated from *A. vinelandii* was added to 6 µM of GF17 apo‐yAnfDK or 12 µM of GF15 or GF19 apo‐yAnfDK. Positive control reactions were carried with 11 µM of *A. vinelandii* apo‐NifDK. The activity of reconstituted yAnfDK was determined by the acetylene reduction assay after addition of AvNifH and ATP‐regenerating mixture. Data represent mean ± SD (*n* = 6) for each yAnfDK.

The yAnfDK protein isolated from GF15, GF17 and GF19 cells was incubated with FeMo‐co isolated from *A*.* vinelandii*. Apo‐NifDK purified from *A*.* vinelandii* was used as control of FeMo‐co reconstitution. The unavailability of FeFe‐co in our laboratory and the functional equivalence between FeFe‐co and FeMo‐co (Harris *et al*., 2018) were the reasons to use FeMo‐co in this assay. Previous results have shown the incorporation of FeMo‐co into apo‐AnfDGK to form a hybrid nitrogenase able to reduce acetylene to ethylene and ethane (Gollan *et al*., 1993). Similarly, apo‐NifDK reconstitution with FeV‐co forms a hybrid nitrogenase that can reduce acetylene to both ethylene and ethane (Smith *et al*., 1988). Activation of yAnfDK by FeMo‐co was then determined by the acetylene reduction assay following addition of excess AvNifH and ATP regeneration mixture (Shah and Brill, 1977; Allen *et al*., 1993). FeMo‐co insertion into 6 µM of yAnfDK isolated from GF17 strain resulted in reconstituted FeFe protein with specific activity of 300 nmol of ethylene formed·min^‐1^·mg of yAnfDK^‐1^ (Fig. 5). Moreover, 12 µM of yAnfDK protein isolated from GF15 and GF19 rendered 60 and 140 nmol of ethylene formed·min^‐1^·mg of yAnfDK^‐1^ respectively. The variability of specific activities shown in Fig. 5 was probably due to the different affinity tags used. These results demonstrate that the yAnfDK present in mitochondria is in fact an apo‐protein readily activated by the simple addition of active‐site cofactor.

## Discussion

Given the genetic simplicity of the Fe‐only nitrogenase, expression of its four structural genes *anfHDGK* could be sufficient to render active Fe protein and cofactor‐activatable FeFe protein. Therefore, we targeted the AnfH, AnfD, AnfG and AnfK polypeptides to the mitochondrial matrix of *S*.* cerevisiae*, with or without the *A*.* vinelandii* [Fe‐S] cluster assembly proteins NifU and NifS. The most salient result of this study is that yAnfDK produced in mitochondria could be activated *in vitro* by the simple addition of FeMo‐co. This activation was possible because FeFe‐co and FeMo‐co are structurally and functionally equivalent (Krahn *et al*., 2002; Harris *et al*., 2018). This activation implies that the yAnfDK complex carries the P‐clusters (or their equivalent in the FeFe protein) and only lacks the active‐site cofactor. Thus, NifU and NifS are not essential for yAnfDK P‐cluster formation in mitochondria.

Fully matured FeFe protein is a heterohexamer of AnfD_2_G_2_K_2_ subunit composition (Schneider and Müller, 2004; O’Carroll and Santos, 2011). However, the yAnfDK protein complex enriched from *S*.* cerevisiae* protein extracts lacked the yAnfG subunit suggesting that yAnfG was either lost during the purification process or that it was never part of a yAnfDGK complex. The complex might be unstable in yeast or require additional components for stability. One obvious component missing is the active‐site FeFe cofactor. The presence of AnfG is relevant because *A*.* vinelandii* strains with altered *anfG* gene expression were shown unable to grow diazotrophically albeit exhibiting acetylene reduction activity (Waugh *et al*., 1995). Similarly, removing *anfG* from the engineered Fe‐only nitrogenase in *E*.* coli* decreased acetylene reduction and eliminated N_2_ fixation activity (Yang *et al*., 2014 *)*.

The Fe protein activity of purified yAnfH preparations was similar to that of mitochondria‐targeted yNifH (López‐Torrejón *et al*., 2016), which is not surprising due to their high (61%) amino acid sequence conservation (Joerger *et al*., 1989). In contrast to yNifH, yAnfH maturation to render active Fe protein was independent of the ancillary protein NifM. Surprisingly, lower yAnfH Fe protein activity was observed when coexpressing NifU and NifS in addition to yAnfHDGK (strain GF16). This study confirms that mitochondria endogenous [Fe‐S] cluster assembly proteins, probably Isu1/Isu2 and Nfs1 (Lill, 2009), are able to produce [Fe_4_S_4_] clusters for yAnfH as they do for yNifH (López‐Torrejón *et al*., 2016). However, because NifU and NifS have been shown essential for yNifB activity (Burén *et al*., 2019), the native mitochondria [Fe‐S] cluster assembly machinery would not be able to replace the function of NifU and NifS in a completely engineered nitrogenase.

In summary, this study demonstrates that expression and targeting of yAnfHDGK proteins to the mitochondrial matrix of *S*.* cerevisiae* is possible and that all proteins appear to be stable. Both structural components can be purified by affinity chromatography. While the Fe protein (yAnfH) was active as‐isolated, the yAnfG subunit was not found as part of the FeFe protein, which existed as apo‐yAnfDK complex. Importantly, apo‐yAnfDK could be activated upon FeMo‐co insertion indicating that it contained the P‐clusters. Future efforts should focus on engineering FeFe‐co assembly in *S*.* cerevisiae* to obtain fully mature nitrogenase. A nitrogen‐fixing yeast could have industrial application as ammonium factory or, by coupling to glutamate dehydrogenase, a glutamate factory.

## Experimental procedures

### Strain growth and culture media

*E*.* coli* DH5α was used for storage and amplification of yeast expression vectors. *E*.* coli* strains were grown at 37ºC in Luria–Bertani medium supplemented with ampicillin when necessary.

*Saccharomyces cerevisiae* W303‐1a (*MAT*a [*leu2‐3,112 trp1‐1 can1‐100 ura3‐1 ade2‐1 his3‐11,15*]) and derivative strains, constructed herein, were grown at 28ºC in flasks at 200 rpm in synthetic dropout (SD) medium composed of 1.9 g l^‐1^ yeast nitrogen base, 5 g l^‐1^ ammonium sulfate, 20 g l^‐1^ glucose and Kaiser dropout mixture (Kaiser *et al*., 1994) supplemented with 20 mg l^‐1^ adenine, 40 mg l^‐1^ tryptophan, 40 mg l^‐1^ histidine, 20 mg l^‐1^ uracil or 60 mg l^‐1^ leucine, depending on auxotrophic requirements. To express *anf* and *nif* genes, 20 g l^‐1^ galactose was added to the culture medium when glucose was consumed. In addition, the medium was supplemented with 5 g l^‐1^ yeast extract, 25 g l^‐1^ bactopeptone and 100 mM of ammonium iron (III) citrate.

### DNA constructs and generation of *E. coli* and *S. cerevisiae* strains

*A*.* vinelandii anfH, anfD, anfK* and *anfG* genes were synthesized with codon optimization for *S*.* cerevisiae* (GenScript, Piscataway, NJ, USA) and cloned in‐frame downstream of DNA sequences encoding a *Su9* mitochondria leader peptide together with either a His_10_ tag, a Strep‐tag or a Twin‐Strep‐tag (Westermann and Neupert, 2000). To generate GF13 strain, the *Su9* encoding sequence 5’ of *anfH* was replaced by a *Mam33* encoding sequence. Transformation of *S*.* cerevisiae* strains was carried out according to Chen *et al*. (1992). Generated strains are listed in Table S1.

To overproduce *A*.* vinelandii* AnfH, AnfD, AnfK and AnfG proteins in *E*.* coli*, the corresponding *anfH, anfD, anfK* and *anfG* genes were amplified by PCR and cloned into the pET16b expression vector. *A*.* vinelandii* genomic DNA was used as template, and the following oligonucleotides were used as primers for PCR (*Nde*I and *BamH*I sites included in the oligonucleotides are underlined): 5’‐AAGTCATATGACTCGTAAAGTAGCCATTTAC‐3’ and 5‐AACTGGATCCTCAGTCGGCAATACCGTACTTGAC‐3’ to amplify *anfH*, 5’‐ AAGTCATATGCCGCATCACGAGTTCGAGTGCAGCAAGGT‐3’ and 5’‐AACTGGATCCTCAGCCGACCTTGCGCTCGAA‐3’ to amplify *anfD*, 5’‐ AAGTCATATGACTTGCGAAGTCAAGGAAAAAGGG‐3’ and 5’‐AACTGGATCCTTACCAGACGTTGAGGACCCATTCC‐3’ to amplify *anfK*, 5’‐AAGTCATATGAGTACCGCTTCCGCCGCTGCTGTGG‐3’ and 5’‐AACTGGATCCTTAATAGTGTTTGTCGCTCA‐3’ to amplify *anfG*. The PCR fragments were isolated and cloned into the *Nde*I and *BamH*I sites of pET16b to generate plasmids pN2GLT19 (*anfH*), pN2GLT20 (*anfD*), pN2GLT21 (*anfK*) and pN2GLT22 (*anfG*) respectively.

Yeast and bacterial strains were stored in 20% glycerol at −80°C. Generated strains are listed in Table S1.

### Rabbit polyclonal antibody production

Polyclonal anti‐AnfH, anti‐AnfD, anti‐AnfK and anti‐AnfG antibodies were generated by immunizing rabbits with the corresponding purified antigens. Recombinant AnfH, AnfD, AnfK and AnfG proteins were purified from isopropyl β‐D‐1‐thiogalactopyranoside (IPTG)‐induced cells of *E*.* coli* BL21 carrying plasmids pN2GLT19, pN2GLT20, pN2GLT21 or pN2GLT22 respectively. Purifications were performed by Co^2+^ affinity chromatography under anaerobic conditions (< 0.1 ppm O_2_) using an AKTA Prime FPLC system (GE Healthcare, Wauwatosa, WI, USA) inside an MBraun glovebox (Fig. S3). AnfD protein was recovered from inclusion bodies in *E*.* coli* BL21 (pN2GLT20) cell‐free extracts by solubilization in buffer containing 50 mM Tris–HCl pH 8.0, 150 mM NaCl, 4 M guanidinium chloride and subsequent refolding by dialysis at 4°C for 4 hours. One mg of each pure protein was sent for antibody production (Centro de Investigaciones Biológicas CIB‐CSIC) following a 1‐rabbit 70‐day protocol with 3 antigen boosters. Anf antibodies were titrated by ELISA Díaz‐Perales *et al*. (2003).

### Western blotting

Anf and Nif proteins were separated by SDS‐PAGE transferred to membranes and detected by immunoblotting using rabbit polyclonal antibodies (1:10,000 dilution for anti‐AnfH, and 1:3,000 dilutions for anti‐AnfD, anti‐AnfK and anti‐AnfG) or commercially available anti‐His‐tag (H‐3, sc‐8036; Santa Cruz Biotechnology, Dallas, TX, USA)‐ and anti‐Strep‐tag II (StrepMAB‐Classic, 2‐1507‐001; IBA Lifesciences, Goettingen, Germany)‐specific antibodies. Antigen–antibody complexes were visualized with goat anti‐Rabbit IgG secondary antibody–HRP (H + L, AS09 602; Agrisera, Vännäs, Sweden).

### Expression of AnfH, AnfG, AnfD, AnfK, NifU and NifS in *S. cerevisiae*

Yeast strains were aerobically cultured according to López‐Torrejón *et al*. (2016) in synthetic dropout (SD) medium containing 1.9 g l^‐1^ yeast nitrogen base, 5 g l^‐1^ ammonium sulfate, 20 g l^‐1^ glucose and Kaiser dropout mixture (SC‐His‐Leu‐Trp‐Ura, ForMedium^TM^) supplemented with 20 mg l^‐1^ adenine and 40 mg l^‐1^ tryptophan, 40 mg l^‐1^ histidine, 20 mg l^‐1^ uracil or 60 mg l^‐1^ leucine, depending on auxotrophic requirements, in a 4 l fermentor, during 16 h at 28ºC. Then, the medium was supplemented with 5 g l^‐1^ of yeast extract, 25 g l^‐1^ of bactopeptone and 100 µM of ammonium iron (III) citrate, and culture continued until glucose was consumed, at which time 20 g l^‐1^ galactose and a trace element solution (13 g l^‐1^ CaCl_2_, 2.5 g l^‐1^ Cl_2_Mn, 0.5 g l^‐1^ ZnSO_4_, 1.4 g l^‐1^ Na_2_ MoO_4_, 1.85 g l^‐1^ FeCl_3_, 1 g l^‐1^ H_3_BO_4_, 0.7 g l^‐1^ IK in 2 M HCl) were added to induce *anf* and *nif* gene expression. Cells were harvested under anoxic conditions in the late exponential phase by centrifugation at 4ºC and 5000 rpm for 5 min. Recovered cell paste was frozen and stored into liquid N_2_ until used.

### Growth of yeast strains for mitochondria isolations

*Saccharomyces cerevisiae* strains GF15, GF16, GF17 and GF18 were grown in flasks at 28°C and 200 rpm in synthetic dropout (SD) medium containing 1.9 g l^‐1^ yeast nitrogen base, 5 g l^‐1^ ammonium sulfate, 20 g l^‐1^ glucose and Kaiser dropout mixture (SC‐His‐Leu‐Trp‐Ura, ForMedium^TM^) supplemented with 20 mg l^‐1^ adenine, 40 mg l^‐1^ tryptophan and 20 mg l^‐1^ uracil, as previously described (López‐Torrejón *et al*., 2016). Protein expression was induced by replacing glucose with galactose in the above‐described SD medium, additionally supplemented with 0.1% yeast extract and 1% peptone, for 16 h. Mitochondria isolations were performed as previously described (Diekert *et al*., 2001). Organelle enrichment was verified using tubulin (cytosolic) and HSP60 (mitochondria) marker proteins.

### His‐tagged yAnfH and yAnfDK protein purification

*Saccharomyces cerevisiae* cells (GF13, GF15, GF16, GF17 or GF19 strain) were re‐suspended in anaerobic buffer A containing 100 mM Tris–HCl pH 8.0, 250 mM NaCl, 1 mM phenylmethylsulfonyl fluoride (PMSF), 1 mM leupeptin, 2 mM sodium dithionite (DTH) and 5 µg ml^‐1^ DNase I. Cells were disrupted in an Emulsiflex‐C5 homogenizer (Avestin Inc., Ottawa, ON, Canada) at 30 000 psi (2,070 bar). Cell‐free extracts (CFE) were filtered in 0.2 µm pore size filter (Nalgene Rapid‐Flow; Thermo Scientific, Waltham, MA, USA), after removing cell debris by centrifugation at 70 000 *g* for 1 h at 4ºC, under anaerobic conditions.

The His‐tagged yAnfH and yAnfDGK proteins were partially purified by Co^2+^ affinity chromatography under anaerobic conditions (< 0.1 ppm O_2_) using an AKTA Prime FPLC system (GE Healthcare) inside an MBraun glovebox. CFE from 150 g of cells was loaded at 2 ml min^‐1^ onto a HiTrap IMAC column loaded with Co^2+^ and equilibrated with anaerobic buffer A. After additional washing with 10 column volumes (CV) of buffer A, the proteins were eluted using a linear gradient of up to 70% elution buffer (280 mM imidazole) within 20 CV. Eluted fractions were concentrated using a Vivaspin 500 concentrator (Sartorius, Goettingen, Germany) with cut‐off pore size of 30 kDa and then desalted in PD10 desalting columns (GE Healthcare) equilibrated with anaerobic buffer B containing 50 mM Tris–HCl, pH 8, 300 mM NaCl, 5% v/v glycerol. Purified proteins were frozen and stored in liquid N_2_. Overall protein concentration in the preparations was determined by the BCA assay using BSA as a standard. The amount of partially purified yAnf components was estimated after gel or membrane scanning and comparison of relative intensities against known amounts of standard proteins (BSA for yAnfH and *E*.* coli* produced AnfD and AnfK for yAnfDK) using the ImageJ software (Taylor *et al*., 2013). Average relative densities are shown in Fig. S4.

### Single‐ and Twin‐Strep‐tag yAnfDK protein purification

*Saccharomyces cerevisiae* GF17 and GF19 cells were re‐suspended in anaerobic buffer A containing 100 mM Tris–HCl pH 8.0, 250 mM NaCl, 1 mM PMSF, 1 mM leupeptin, 2 mM DTH and 5 µg ml^‐1^ DNase I. Cells were lysed in an Emulsiflex‐C5 homogenizer (Avestin Inc.) at 30 000 psi (2,070 bar). CFE was obtained by centrifugation at 70 000 *g* for 1 h at 4ºC under anaerobic conditions.

Single‐ or Twin‐Strep‐tagged yAnfDK proteins were purified by Strep‐Tactin Superflow XT affinity chromatography (IBA) under anaerobic conditions (< 0.1 ppm O_2_) using an AKTA Prime FPLC system (GE Healthcare) inside an MBraun glovebox. CFE from 200 g of cells was loaded at 2 ml min^‐1^ onto a Strep‐Tactin Superflow XT column equilibrated with anaerobic buffer A and washed with 10 CV with same buffer. Finally, bound protein was eluted with buffer A containing 50 mM biotin. Co‐eluted yAnfDK proteins were concentrated with a Vivaspin 500 concentrator (Sartorius) with cut‐off pore size of 30 kDa and then desalted in PD10 desalting columns (GE Healthcare) equilibrated with buffer B containing 50 mM Tris–HCl, pH 8, 300 mM NaCl, 5% v/v glycerol. Purified proteins were frozen and stored in liquid N_2_. Overall protein concentration in the preparations was determined by the BCA assay using BSA as a standard. The amount of partially purified yAnfDK was estimated after immunoblot membrane scanning and comparison of relative intensity against known amounts of pure AnfD and AnfK (produced in *E*.* coli*) using ImageJ software (Taylor *et al*., 2013). Average relative densities are shown in Fig. S4.

### Fe protein activity determination

Fe protein activity of yAnfH preparations obtained from *S*.* cerevisiae* GF13, GF15, GF16, GF17 and GF19 strains was routinely analysed by the acetylene reduction assay after addition of excess pure *A*.* vinelandii* NifDK (AvNifDK) and ATP‐regenerating mixture (1.23 mM ATP, 18 mM phosphocreatine, 2.2 mM MgCl_2_, 3 mM DTH and 40 µg of creatine phosphokinase) in 100 mM MOPS pH 7.0 (Shah and Brill, 1973; Emerich and Burris, 1978). To titrate AvNifDK activity with yAnfH, increasing yAnfH to AvNifDK molar ratios were assayed. Positive control reactions were carried out with AvNifH and AvNifDK proteins purified from *A*.* vinelandii* cells under anaerobic conditions as described (Curatti *et al*., 2007).

### In vitro apo‐yAnfDK activation by FeMo‐co isolated from *A. vinelandii*

Apo‐AvNifDK preparations were isolated under anaerobic conditions from *A*.* vinelandii* cells as described (Curatti *et al*., 2007). FeMo‐co was isolated in NMF from purified preparations of *A*.* vinelandii* NifDK as reported in Shah *et al*. (1977).

The apo‐yAnfDK activation assay was carried out by adding 5 µl of isolated FeMo‐co and incubating at 30**°**C for 10 min under anaerobic conditions in 100 mM MOPS pH 7.0 plus 1.2 µg ml^‐1^ BSA. Six or 12 µM apo‐yAnfDK (obtained from GF15, GF17 or GF19 cells) was used in reconstitution reactions. Reactions containing 11 µM of *A*.* vinelandii* apo‐NifDK were used as positive control. The resulting activation of apo‐NifDK or apo‐yAnfDK present in the reaction mixtures was analysed by the acetylene reduction assay after addition of AvNifH and ATP‐regenerating mixture (1.23 mM ATP, 18 mM phosphocreatine, 2.2 mM MgCl_2_, 3 mM DTH and 40 µg of creatine phosphokinase) in a 9 ml stoppered vials with Ar/C_2_H_2_ filling the headspace, at 30**°**C for 30 min, and then quenched by addition of 0.1 ml of 8 M NaOH.

## Funding Information

This work was supported by the Bill and Melinda Gates Foundation Grant OPP1143172 to LMR.

## Conflict of interest

None declared.

## Author contributions

LMR and GLT designed the experiments. GLT and SB conducted the experiments. MV carried out yeast fermentations and some DNA constructs. GLT, SB and LMR analysed data and wrote the manuscript. The authors have no conflict of interest.

## Supporting information


**Fig. S1**. DNA optimized sequences.

**Fig. S2**. Schematic representation of synthetic *anf* and *nif* genes in *S. cerevisia*e GF15, GF16 and GF18.

**Fig. S3**. Purification of AnfH, AnfK, AnfG and AnfD proteins for the generation of polyclonal antibodies.

**Fig. S4**. Quantification of yAnfH and yAnfDK proteins in partially purified fractions.

**Table S1**. List of strains used in this work.
