# Supplementary material for: Biosynthesis of cofactor‐activatable iron‐only nitrogenase in Saccharomyces cerevisiae
Source: Microb Biotechnol. 2021 Jan 28;14(3):1073–83. doi: 10.1111/1751-7915.13758 (PMC8085987; doi:10.1111/1751-7915.13758)
Supplement: Supplementary file 1 — Fig. S1. DNA optimized sequences. [file MBT2-14-1073-s002.docx]

GAL10p::*mlsSu9-anfG* in pESC-His

gcggccgcatggcatccacaagagtattggcatcaagattagcatcacaaatggcagcctca

M A S T R V L A S R L A S Q M A A S

gcaaaagtcgcaagaccagcagttagagtagcacaagtttctaagagaactatccaaaca

A K V A R P A V R V A Q V S K R T I Q T

ggttcaccattgcaaactttgaagagaactcaaatgacatctattgttaacgcaactaca

G S P L Q T L K R T Q M T S I V N A T T

agacaagcttttcaaaagagagcatattcttcaatgtctactgcttcagctgcagctgtt

R Q A F Q K R A Y S S M S T A S A A A V

gttaaacaaaaagttgaagctccagttcatccaatggatgcaagaattgatgaattgaca

V K Q K V E A P V H P M D A R I D E L T

gattacatcatgaagaactgtttatggcaatttcattctagatcatgggatagagaaaga

D Y I M K N C L W Q F H S R S W D R E R

caaaacgcagaaatcttgaagaaaactaaggaattgttgtgtggtgaaccagttgatttg

Q N A E I L K K T K E L L C G E P V D L

tctacttcacatgatagatgttactgggttgatgcagtttgtttggctgatgattacaga

S T S H D R C Y W V D A V C L A D D Y R

gaacattacccatggatcaactctatgtcaaaggaagaaatcggttcattgatgcaaggt

E H Y P W I N S M S K E E I G S L M Q G

ttaaaagatagaatggactacttgactatcactggttccttgaatgaagaattatctgac

L K D R M D Y L T I T G S L N E E L S D

aaacactattaaatcgat

K H Y *

GAL1p::*mlsSu9*-*his_10_*-*anfH* in pESC-His

ggatccatggcctccactcgtgtcctcgcctctcgcctggcctcccagatggctgcttcc

M A S T R V L A S R L A S Q M A A S

gccaaggttgcccgccctgctgtccgcgttgctcaggtcagcaagcgcaccatccagact

A K V A R P A V R V A Q V S K R T I Q T

ggctcccccctccagaccctcaagcgcacccagatgacctccatcgtcaacgccaccacc

G S P L Q T L K R T Q M T S I V N A T T

cgccaggctttccagaagcgcgcctactcttccatgcatcatcaccatcaccatcaccat

R Q A F Q K R A Y S S M **H H H H H H H H**

caccatacaagaaaggttgctatctatggtaaaggtggaattggaaagagtactacaacc

**H H** T R K V A I Y G K G G I G K S T T T

caaaatactgctgcagctttggcatacttccatgataaaaaggttttcatccacggttgc

Q N T A A A L A Y F H D K K V F I H G C

gatccaaaagctgactccactagattgatattaggtggaaagcctcaagaaacacttatg

D P K A D S T R L I L G G K P Q E T L M

gatatgttgagggacaaaggtgctgaaaagatcacaaacgatgacgttattaagaaagga

D M L R D K G A E K I T N D D V I K K G

tttcttgatatacagtgtgtggaatctggtggaccagagcctggtgttggatgcgcaggt

F L D I Q C V E S G G P E P G V G C A G

agaggagtgatcaccgctatagatttgatggaagagaacggtgcttacactgatgactta

R G V I T A I D L M E E N G A Y T D D L

gacttcgttttctttgatgtgcttggagacgttgtgtgtggtggattcgcaatgccaata

D F V F F D V L G D V V C G G F A M P I

agagatggtaaagctcaagaagtttatattgtggcatcaggagagatgatggctatctat

R D G K A Q E V Y I V A S G E M M A I Y

gcagctaacaacatctgtaagggtttggttaagtacgctaagcagtctggagtgaggtta

A A N N I C K G L V K Y A K Q S G V R L

ggtggaattatctgcaactcaagaaaggttgatggtgaaagggagtttttagaagagttc

G G I I C N S R K V D G E R E F L E E F

accgcagctattggaactaaaatgatccattttgttccaagagacaatattgtgcaaaag

T A A I G T K M I H F V P R D N I V Q K

gcagaattcaataagaaaactgttacagaattcgcacctgaagagaatcaggctaaagaa

A E F N K K T V T E F A P E E N Q A K E

tatggagagcttgctaggaagataattgaaaacgatgagttcgtgatccctaagcctttg

Y G E L A R K I I E N D E F V I P K P L

actatggatcagttagaagacatggttgtgaagtatggtatcgcagattgagtcgac

T M D Q L E D M V V K Y G I A D *

GAL10p:: *mlsSu9*-*anfK* in pESC-Leu

gcggccgcatggcttccaccagagtattagcatccagattagcatcccaaatggcagcatca

M A S T R V L A S R L A S Q M A A S

gcaaaagtagcaagaccagcagtaagagtcgcacaagtttcaaagagaactatccaaaca

A K V A R P A V R V A Q V S K R T I Q T

ggttctccattgcaaactttgaagagaactcaaatgacatcaatcgttaacgcaactaca

G S P L Q T L K R T Q M T S I V N A T T

agacaagcttttcaaaagagagcatactcttcaatgacatgtgaagttaaggaaaagggt

R Q A F Q K R A Y S S M T C E V K E K G

agagttggtactattaatccaatttttacatgtcaaccagctggtgcacaattcgtttct

R V G T I N P I F T C Q P A G A Q F V S

atcggtattaaagattgtattggtattgttcatggtggtcaaggttgtgttatgttcgtt

I G I K D C I G I V H G G Q G C V M F V

agattgattttctctcaacattacaaagaatcttttgaattggcttcttcatctttacat

R L I F S Q H Y K E S F E L A S S S L H

gaagatggtgctgtttttggtgcatgtggtagagttgaagaagcagttgatgttttgtta

E D G A V F G A C G R V E E A V D V L L

tcaagatacccagatgttaaggttgttccaatcatcactacatgttctactgaaatcatt

S R Y P D V K V V P I I T T C S T E I I

ggtgacgatgttgatggtgttattaagaaattgaacgaaggtttgttgaaagaaaagttc

G D D V D G V I K K L N E G L L K E K F

ccagatagagaagttcatttgattgctatgcatacaccatcttttgttggttcaatgatt

P D R E V H L I A M H T P S F V G S M I

tctggttacgatgttgcagttagagatgttgttagacatttcgctaagagagaagcacca

S G Y D V A V R D V V R H F A K R E A P

aacgataaaattaatttgttgactggttgggttaatccaggtgacgttaaagaattgaag

N D K I N L L T G W V N P G D V K E L K

catttgttgggtgaaatggatatcgaagctaacgttttgttcgaaatcgaatcattcgat

H L L G E M D I E A N V L F E I E S F D

tctccaattttaccagatggttcagcagtttctcatggtaacactacaatcgaagatttg

S P I L P D G S A V S H G N T T I E D L

atcgatactggtaatgctagagcaacattcgctttgaacagatacgaaggtacaaaagct

I D T G N A R A T F A L N R Y E G T K A

gcagaatacttgcaaaagaaattcgaaatcccagctatcatcggtccaactccaatcggt

A E Y L Q K K F E I P A I I G P T P I G

attagaaacacagatattttcttgcaaaatttgaagaaagctactggtaaaccaattcca

I R N T D I F L Q N L K K A T G K P I P

caatcattagctcatgaaagaggtgttgcaattgatgctttggcagatttgacacatatg

Q S L A H E R G V A I D A L A D L T H M

tttttggctgaaaagagagttgctatctatggtgcaccagatttggttattggtttagca

F L A E K R V A I Y G A P D L V I G L A

gaattctgtttggatttggaaatgaagccagttttgttgttgttgggtgacgataactct

E F C L D L E M K P V L L L L G D D N S

aagtacgttgatgatccaagaattaaagctttgcaagaaaacgttgattacggtatggaa

K Y V D D P R I K A L Q E N V D Y G M E

atcgttactaacgcagatttctgggaattggaaaacagaattaaaaatgaaggtttggaa

I V T N A D F W E L E N R I K N E G L E

ttggatttgatcttgggtcattcaaagggtagattcatttctatcgattacaacatccca

L D L I L G H S K G R F I S I D Y N I P

atgttaagagttggttttccaacttacgatagagctggtttgtttagatatccaacagtt

M L R V G F P T Y D R A G L F R Y P T V

ggttacggtggtgctatttggttagcagaacaaatggctaatactttatttgccgatatg

G Y G G A I W L A E Q M A N T L F A D M

gaacacaagaagaacaaggaatgggtattgaatgtatggtaaactagt

E H K K N K E W V L N V W *

GAL1p:: *mlsSu9*-***his_10_****-anfD* in pESC-Leu

ggatccatggcatcaaccagagtattggcttcaagattggcatcacaaatggcagcatca

M A S T R V L A S R L A S Q M A A S

gcaaaagtagcaagaccagcagttagagtcgcacaagtttctaagagaactatccaaaca

A K V A R P A V R V A Q V S K R T I Q T

ggttcaccattgcaaacattgaagagaactcaaatgacatctatcgttaacgctactaca

G S P L Q T L K R T Q M T S I V N A T T

agacaagcatttcaaaagagagcttattcttcaatgcatcaccaccatcaccaccatcat

R Q A F Q K R A Y S S M **H H H H H H H H**

catcatccacatcatgaattcgaatgttcaaaagttattccagaaagaaagaaacatgct

**H H** P H H E F E C S K V I P E R K K H A

gttattaaaggtaaaggtgaaacattggctgatgcattaccacaaggttatttgaacact

V I K G K G E T L A D A L P Q G Y L N T

atcccaggttctatctcagaaagaggttgtgcttactgtggtgcaaaacatgttattggt

I P G S I S E R G C A Y C G A K H V I G

acaccaatgaaggatgttatccatatctctcatggtccagttggttgtacttacgataca

T P M K D V I H I S H G P V G C T Y D T

tggcaaactaagagatacatctcagataacgataacttccaattgaagtacacatacgca

W Q T K R Y I S D N D N F Q L K Y T Y A

actgatgttaaggaaaagcatattgtttttggtgctgaaaagttgttgaagcaaaacatc

T D V K E K H I V F G A E K L L K Q N I

atcgaagcttttaaagcatttccacaaattaaaagaatgactatctatcaaacatgtgct

I E A F K A F P Q I K R M T I Y Q T C A

actgcattaattggtgacgatattaatgctattgcagaagaagttatggaagaaatgcca

T A L I G D D I N A I A E E V M E E M P

gaagttgatatcttcgtttgtaactctccaggttttgcaggtccatctcaatcaggtggt

E V D I F V C N S P G F A G P S Q S G G

catcataagattaatatcgcttggatcaaccaaaaagttggtacagttgaaccagaaatc

H H K I N I A W I N Q K V G T V E P E I

actggtgaccatgttattaattacgttggtgaatacaacattcaaggtgaccaagaagtt

T G D H V I N Y V G E Y N I Q G D Q E V

atggttgattacttcaagagaatgggtattcaagttttgtctacttttacaggcaatggt

M V D Y F K R M G I Q V L S T F T G N G

tcatacgatggtttaagagctatgcatagagcacatttgaatgttttagaatgtgctaga

S Y D G L R A M H R A H L N V L E C A R

tctgcagaatacatctgtaacgaattgagagttagatacggtattccaagattggatatc

S A E Y I C N E L R V R Y G I P R L D I

gatggtttcggttttaaaccattggcagattcattgagaaagatcggcatgtttttcggt

D G F G F K P L A D S L R K I G M F F G

atcgaagatagagctaaggcaatcatcgatgaagaagttgctagatggaaaccagaattg

I E D R A K A I I D E E V A R W K P E L

gattggtacaaggaaagattgatgggtaaaaaggtttgtttgtggccaggtggttctaaa

D W Y K E R L M G K K V C L W P G G S K

ttatggcattgggctcatgtcattgaagaagaaatgggtttgaaggttgtttcagtttac

L W H W A H V I E E E M G L K V V S V Y

acaaagttcggtcatcaaggtgacatggaaaaaggtattgcaagatgtggtgaaggtact

T K F G H Q G D M E K G I A R C G E G T

ttggctatcgatgatccaaacgaattggaaggtttagaagcattggaaatgttgaagcca

L A I D D P N E L E G L E A L E M L K P

gatatcatcttgactggtaaaagaccaggtgaagttgctaagaaagttagagttccatat

D I I L T G K R P G E V A K K V R V P Y

ttgaatgctcatgcatatcataatggtccatacaaaggttttgaaggttgggttagattc

L N A H A Y H N G P Y K G F E G W V R F

gcaagagatatctataacgctatctattctccaatccatcaattgtcaggtatcgatatc

A R D I Y N A I Y S P I H Q L S G I D I

acaaaggataacgcaccagaatggggtaatggttttagaactagacaaatgttgtctgat

T K D N A P E W G N G F R T R Q M L S D

ggtaatttgtcagatgctgttagaaactctgaaacattgagacaatacactggtggttac

G N L S D A V R N S E T L R Q Y T G G Y

gacagtgtatctaagttgagagaaagagaatatccagcatttgaaagaaaagttggttga

D S V S K L R E R E Y P A F E R K V G -

Gtcgac

GAL1p:: *mlsSu9*-***streptag****-anfD* in pESC-Leu

ggatccatggcatcaaccagagtattggcttcaagattggcatcacaaatggcagcatca

M A S T R V L A S R L A S Q M A A S

gcaaaagtagcaagaccagcagttagagtcgcacaagtttctaagagaactatccaaaca

A K V A R P A V R V A Q V S K R T I Q T

ggttcaccattgcaaacattgaagagaactcaaatgacatctatcgttaacgctactaca

G S P L Q T L K R T Q M T S I V N A T T

agacaagcatttcaaaagagagcttattcttcaatggcctctgcatggagtcatcctcag

R Q A F Q K R A Y S S **M A S A W S H P Q**

tttgagaaaggtggaggttcaggtggtggaagcggtggatctgcttggtcacatccacaa

**F E K G G G S G G G S G G S A W S H P Q**

tttgaaaaaccacatcatgaattcgaatgttcaaaagttattccagaaagaaagaaacat

**F E K** P H H E F E C S K V I P E R K K H

gctgttattaaaggtaaaggtgaaacattggctgatgcattaccacaaggttatttgaac

A V I K G K G E T L A D A L P Q G Y L N

actatcccaggttctatctcagaaagaggttgtgcttactgtggtgcaaaacatgttatt

T I P G S I S E R G C A Y C G A K H V I

ggtacaccaatgaaggatgttatccatatctctcatggtccagttggttgtacttacgat

G T P M K D V I H I S H G P V G C T Y D

acatggcaaactaagagatacatctcagataacgataacttccaattgaagtacacatac

T W Q T K R Y I S D N D N F Q L K Y T Y

gcaactgatgttaaggaaaagcatattgtttttggtgctgaaaagttgttgaagcaaaac

A T D V K E K H I V F G A E K L L K Q N

atcatcgaagcttttaaagcatttccacaaattaaaagaatgactatctatcaaacatgt

I I E A F K A F P Q I K R M T I Y Q T C

gctactgcattaattggtgacgatattaatgctattgcagaagaagttatggaagaaatg

A T A L I G D D I N A I A E E V M E E M

ccagaagttgatatcttcgtttgtaactctccaggttttgcaggtccatctcaatcaggt

P E V D I F V C N S P G F A G P S Q S G

ggtcatcataagattaatatcgcttggatcaaccaaaaagttggtacagttgaaccagaa

G H H K I N I A W I N Q K V G T V E P E

atcactggtgaccatgttattaattacgttggtgaatacaacattcaaggtgaccaagaa

I T G D H V I N Y V G E Y N I Q G D Q E

gttatggttgattacttcaagagaatgggtattcaagttttgtctacttttacaggcaat

V M V D Y F K R M G I Q V L S T F T G N

ggttcatacgatggtttaagagctatgcatagagcacatttgaatgttttagaatgtgct

G S Y D G L R A M H R A H L N V L E C A

agatctgcagaatacatctgtaacgaattgagagttagatacggtattccaagattggat

R S A E Y I C N E L R V R Y G I P R L D

atcgatggtttcggttttaaaccattggcagattcattgagaaagatcggcatgtttttc

I D G F G F K P L A D S L R K I G M F F

ggtatcgaagatagagctaaggcaatcatcgatgaagaagttgctagatggaaaccagaa

G I E D R A K A I I D E E V A R W K P E

ttggattggtacaaggaaagattgatgggtaaaaaggtttgtttgtggccaggtggttct

L D W Y K E R L M G K K V C L W P G G S

aaattatggcattgggctcatgtcattgaagaagaaatgggtttgaaggttgtttcagtt

K L W H W A H V I E E E M G L K V V S V

tacacaaagttcggtcatcaaggtgacatggaaaaaggtattgcaagatgtggtgaaggt

Y T K F G H Q G D M E K G I A R C G E G

actttggctatcgatgatccaaacgaattggaaggtttagaagcattggaaatgttgaag

T L A I D D P N E L E G L E A L E M L K

ccagatatcatcttgactggtaaaagaccaggtgaagttgctaagaaagttagagttcca

P D I I L T G K R P G E V A K K V R V P

tatttgaatgctcatgcatatcataatggtccatacaaaggttttgaaggttgggttaga

Y L N A H A Y H N G P Y K G F E G W V R

ttcgcaagagatatctataacgctatctattctccaatccatcaattgtcaggtatcgat

F A R D I Y N A I Y S P I H Q L S G I D

atcacaaaggataacgcaccagaatggggtaatggttttagaactagacaaatgttgtct

I T K D N A P E W G N G F R T R Q M L S

gatggtaatttgtcagatgctgttagaaactctgaaacattgagacaatacactggtggt

D G N L S D A V R N S E T L R Q Y T G G

tacgacagtgtatctaagttgagagaaagagaatatccagcatttgaaagaaaagttggt

Y D S V S K L R E R E Y P A F E R K V G

tgagtcgac
