# Supplementary figures and images for: Biosynthesis of cofactor‐activatable iron‐only nitrogenase in Saccharomyces cerevisiae
Source: Microb Biotechnol. 2021 Jan 28;14(3):1073–83. doi: 10.1111/1751-7915.13758 (PMC8085987; doi:10.1111/1751-7915.13758)

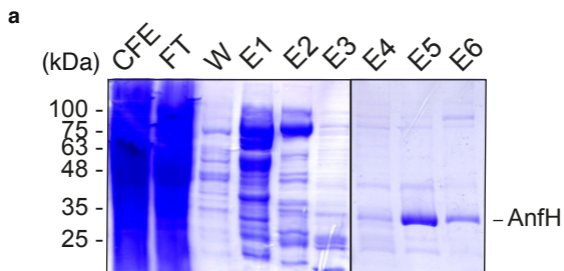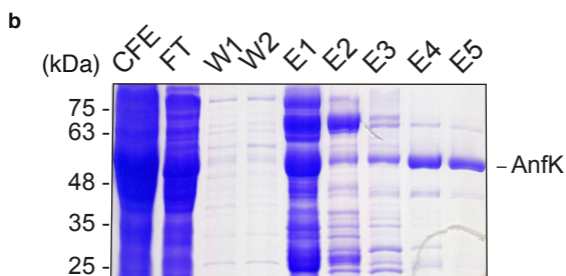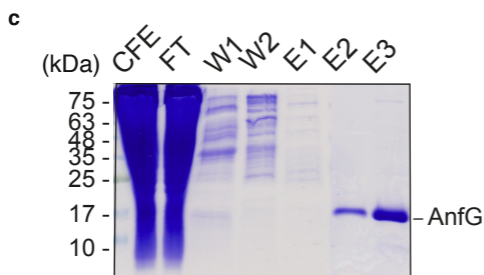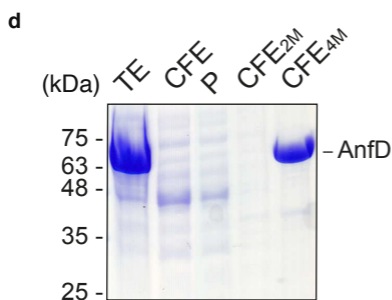

Supplement: Supplementary file 3 — Fig. S3. Purification of AnfH, AnfK, AnfG and AnfD proteins for the generation of polyclonal antibodies. [file MBT2-14-1073-s003.pdf]
