# Supplementary material for: Biosynthesis of cofactor‐activatable iron‐only nitrogenase in Saccharomyces cerevisiae
Source: Microb Biotechnol. 2021 Jan 28;14(3):1073–83. doi: 10.1111/1751-7915.13758 (PMC8085987; doi:10.1111/1751-7915.13758)
Supplement: Supplementary file 5 — Table S1. List of strains used in this work. [file MBT2-14-1073-s004.docx]

**Supplementary Table 1.** **List of strains used in this work.**

| **Strain** | **Genotype** | **Source** |
| --- | --- | --- |
| ***A. vinelandii*** |  |  |
| DJ | Wild type | Dr. Dennis Dean |
| ***E. coli*** |  |  |
| DH5α | F- φ80Δ*lacZM15* Δ(*lacZYA-argF)U169 deoP recA1 endA1 hsdR17* (r_K_- m_K_-) | Sambrook 2001 |
| BL21(DE3) | F- (*ompT* r_B_-m_B_-) | Novagene |
| BL21 pN2GLT19 | F- (*ompT* r_B_-m_B_-), T7l*_ac_*::*his*-*anfH* | This work |
| BL21 pN2GLT20 | F- (*ompT* r_B_-m_B_-), T7l*_ac_*::*his*-*anfD* | This work |
| BL21 pN2GLT21 | F- (*ompT* r_B_-m_B_-), T7l*_ac_*::*his*-*anfK* | This work |
| BL21 pN2GLT22 | F- (*ompT* r_B_-m_B_-), T7l*_ac_*::*his*-*anfG* | This work |
| ***S. cerevisiae*** |  |  |
| W303-1a | *MAT*a {*leu2-3, 112 trp1-1 can1-100 ura3-1 ade2-1 his3-11,15*} | ATCC |
| GF13 | W303-1a GAL1p::*mlsmam33*-*his_10_*-*anfH* in pESC-His and GAL1p::*mlsSu9-nifU* and GAL10p::*mlsSu9-nifS* in pESC-Ura | This work |
| GF14 | W303-1a GAL1p:: *mlsSu9*-*his_10_*-*anfH* and GAL10p::*mlsSu9-anfG* in pESC-His | This work |
| GF15 | W303-1a GAL1p::*mlsSu9*-*his_10_*-*anfH* and GAL10p::*mlsSu9-anfG* in pESC-His and GAL1p:: *mlsSu9*- *his_10_*-*anfD* and GAL10p::*mlsSu9-anfK* in pESC-Leu | This work |
| GF16 | W303-1a GAL1p::*mlsSu9*-*his_10_*-*anfH* and GAL10p::*mlsSu9-anfG* in pESC-His and GAL1p:: *mlsSu9*- *his_10_*-*anfD*, GAL10p::*mlsSu9-anfK* in pESC-Leu and GAL1p::*mlsSu9-nifU* and GAL10p::*mlsSu9-nifS* in pESC-Ura | This work |
| GF17 | W303-1a GAL1p::*mlsSu9*-*his_10_*-*anfH* and GAL10p::*mlsSu9-anfG* in pESC-His and GAL1p:: *mlsSu9-twin*-*streptag*-*anfD* and GAL10p::*mlsSu9-anfK* in pESC-Leu | This work |
| GF18 | W303-1a GAL1p::*mlsSu9*-*his_10_*-*anfH* and GAL10p::*mlsSu9-anfG* in pESC-His and GAL1p:: *mlsSu9*-*twin*-*streptag*-*anfD*, GAL10p::*mlsSu9-anfK* in pESC-Leu and GAL1p::*mlsSu9-nifU* and GAL10p::*mlsSu9-nifS* in pESC-Ura | This work |
| GF19 | W303-1a GAL1p::*mlsSu9*-*his_10_*-*anfH* and GAL10p::*mlsSu9-anfG* in pESC-His and GAL1p:: *mlsSu9*-*streptag*-*anfD* and GAL10p::*mlsSu9-anfK* in pESC-Leu | This work |
| GF20 | W303-1a GAL1p::*mlsSu9*-*his_10_*-*anfH* and GAL10p::*mlsSu9-anfG* in pESC-His and GAL1p:: *mlsSu9*-*streptag*-*anfD* and GAL10p::*mlsSu9-anfK* in pESC-Leu and GAL1p::*mlsSu9-nifU* and GAL10p::*mlsSu9-nifS* in pESC-Ura | This work |
