# Supplementary material for: Biosynthesis of cofactor‐activatable iron‐only nitrogenase in Saccharomyces cerevisiae
Source: Microb Biotechnol. 2021 Jan 28;14(3):1073–83. doi: 10.1111/1751-7915.13758 (PMC8085987; doi:10.1111/1751-7915.13758)
Supplement: Supplementary file 6 [file MBT2-14-1073-s005.docx]

**Fig. S1**. **DNA optimized sequences.** Yeast codon optimized sequences of the A. vinelandii anfH, anfD, anfG, and anfK genes synthetized by GenScript (USA). Sequences include the su9 mitochondria leader sequence 5’ of each gene and the restriction sites used for cloning into pESC vectors (underlined).

**Fig. S2. Schematic representation of synthetic anf and nif genes in S. cerevisiae GF15, GF16 and GF18**. Scheme shows relative positions of mitochondria leading sequences and purification tags. **a**, GF15 strain mlsSu9-his_10_-anfH, mlsSu9-anfG, mlsSu9- his_10_-anfD and mlsSu9-anfK; **b**, GF16 strain mlsSu9-his_10_-anfH, mlsSu9-anfG, mlsSu9- his_10_-anfD and mlsSu9-anfK, mlsSu9-nifU and mlsSu9-nifS; **c**, GF18 strain mlsSu9-his_10_-anfH, mlsSu9-anfG, mlsSu9-twin-streptag-anfD, mlsSu9-anfK, mlsSu9-nifU and mlsSu9-nifS.

**Fig. S3. Purification of AnfH, AnfK, AnfG and AnfD proteins for the generation of polyclonal antibodies.** E. coli cultures were used to express and purify His-tagged AnfH, AnfK, AnfG and AnfD proteins. Coomassie staining of SDS gels showing the purification process of AnfH (**a**), AnfK (**b**), and AnfG (**c**) using HiTrap chelating HP chromatography.  CFE, soluble cell-free extract; FT, Flow-through fraction; W1, protein fraction eluted after washing with binding buffer; W2, protein fraction eluted after washing with binding buffer supplemented with 80 mM imidazole; E1, E2, E3 of  protein fractions eluted after applying a gradient of imidazole (80-240 mM) in binding buffer. (**d**) Insoluble AnfD protein was recovered from inclusion bodies of E. coli cell-free extracts by solubilization in buffer containing 50 mM Tris-HCl pH 8.0, 150 mM NaCl, 4 M guanidinium hydrochloride and subsequent refolding by dialysis at 4°C for 4 hours. The dialyzed and refolded AnfD protein was recovered with high efficiency and mainly free from contaminants (CFE4M fraction). TE, total cell protein; CFE, soluble cell-free extract; P, pellet after solubilization with guanidine hydrochloride. Approximately 1 mg of each purified protein was used as immunogen in rabbits. The final bleed of each rabbit was stored and used for immunoblots after titration.

**Fig. S4. Quantification of yAnfH and yAnfDK proteins in partially purified fractions**. **a**, Coomassie staining of SDS gel of His-tagged yAnfH protein isolated from GF15 strain (left) and BSA standards (right, 2 mg and 4 mg). **b**, Immunoblot analysis of preparations enriched in yAnfDK proteins from GF15, GF17 and GF19 (HisAnfDK, TSAnfDK and TSAnfDK proteins, respectively). Known amounts of AnfDK polypeptides purified from E. coli were used as standards (25, 50 and 100 ng). Average relative densities after scanning with ImageJ software are shown below the gel and membranes.
